# Development of a Portable Detection Method for Enteric Viruses from Ambient Air and Its Application to a Wastewater Treatment Plant

**DOI:** 10.3390/pathogens8030131

**Published:** 2019-08-24

**Authors:** Koichi Matsubara, Hiroyuki Katayama

**Affiliations:** Department of Urban Engineering, School of Engineering, The University of Tokyo, Tokyo 113-8656, Japan

**Keywords:** virus, aerosols, pathogenic microorganisms, real-time PCR

## Abstract

The ambient air from wastewater treatment plants has been considered as a potential source of pathogenic microorganisms to cause an occupational risk for the workers of the plants. Existing detection methods for enteric viruses from the air using a liquid as the collection medium therefore require special care to handle on-site. Knowledge accumulation on airborne virus risks from wastewater has been hindered by a lack of portable and handy collection methods. Enteric viruses are prevalent at high concentrations in wastewater; thus, the surrounding air may also be a potential source of viral transmission. We developed a portable collection and detection method for enteric viruses from ambient air and applied it to an actual wastewater treatment plant in Japan. Materials of the collection medium and eluting methods were optimized for real-time polymerase chain reaction-based virus quantification. The method uses a 4 L/min active air sampler, which is capable of testing 0.7–1.6 m^3^ air after 3–7 h sampling with a detection limit of 10^2^ copies/m^3^ air in the field. Among 16 samples collected at five to seven locations in three sampling trials (November 2007–January 2008), 56% (9/16) samples were positive for norovirus (NV) GII, with the highest concentration of 3.2 × 10^3^ copies/m^3^ air observed at the sampling point near a grit chamber. Adenoviruses (4/16), NV GI (6/16), FRNA bacteriophages GIII (3/16), and enteroviruses (3/16) were also detected but at lower concentrations. The virus concentration in the air was associated with that of the wastewater at each process. The results imply that the air from the sewer pipes or treatment process is contaminated by enteric viruses and thus special attention is needed to avoid accidental ingestion of viruses via air.

## 1. Introduction

Wastewater treatment plants are considered as potential sources of pathogenic bioaerosols [1]. Several studies have demonstrated that high amounts of microorganisms are present not only in the wastewater but also in bioaerosols generated from wastewater treatment processes [2,3,4]. Bioaerosols are suspected to have adverse health effects on the neighboring residents of wastewater treatment processes [2] or wastewater treatment plant (WWTP) workers [5]; however, there are limited studies about the detection of enteric viruses from bioaerosols [2,6]. These studies used cell culture assays and detected enterovirus (EV) and reovirus which did not reflect the actual occurrence of viruses because most viruses are practically difficult to propagate in cell lines. Moreover, the research field for bioaerosol monitoring has predominately focused on the detection of fungal spores and bacteria, where the analysis of samples depends on total-count or culture techniques.

Enteric viruses are shed in the feces of infected patients; thus, they are frequently detected at high concentrations in wastewater samples [7]. They are transmitted mainly through the fecal–oral route via contaminated food and water, but some epidemiological reports have shown that enteric viruses, especially noroviruses (NVs), can cause outbreaks through aerosols released from vomit [8,9]. Quantitative polymerase chain reaction (qPCR) has been widely used to detect enteric viruses in wastewater because some enteric viruses such as human NVs cannot routinely be propagated in cell lines [10]. Furthermore, the PCR assays have the advantages of specificity, sensitivity, and rapidity in the detection; hence, this can be a reliable method for detecting viruses in bioaerosols. 

A previous study detected noroviruses from the air using dust filter (PTFE filter with the pore size 1 µm), while the method was not optimized for virus detection and the detection rate was low (only one in four field samples) [4]. Another recent study detected rotavirus and adenovirus (AdV) quantitatively with a liquid collector and cascade sampler using PCR [11]. However, knowledge is limited partly due to the complicated sampling method. The lack of a portable collection method hampers knowledge accumulation on airborne virus risks from wastewater. The reliable existing method uses liquid for collection [12], but this is not convenient for sampling as it requires a regular power supply (AC 100–200 V), which is not always available in the field or specific locations of WWTPs.

Also, the liquid medium requires special care to be handled on-site to avoid contamination. Collection media for air sampling is vulnerable to contamination since viruses that originate from wastewater are abundant in the environment in WWTPs. Operation at an unevenly leveled location or transportation from the field to the laboratory can also cause the liquid to spill from its container. There was also an attempt to use membranes for sampling in previous literature [6]. However, it was not optimized for detecting viruses and for PCR detection processes. Therefore, it is important to develop and test a handy, battery-driven sampling method using a membrane optimized for qPCR.

The objective of this study was to develop a mobile sampling device and sampling procedure for the detection of enteric viruses in bioaerosols by PCR-based assay, and to apply the method at an actual WWTP. In this course, we developed a novel mobile sampling method and verified it via field sampling at an actual WWTP.

## 2. Materials and Methods

### 2.1. Development of Collection Method and Laboratory Evaluation

**Figure 1:**
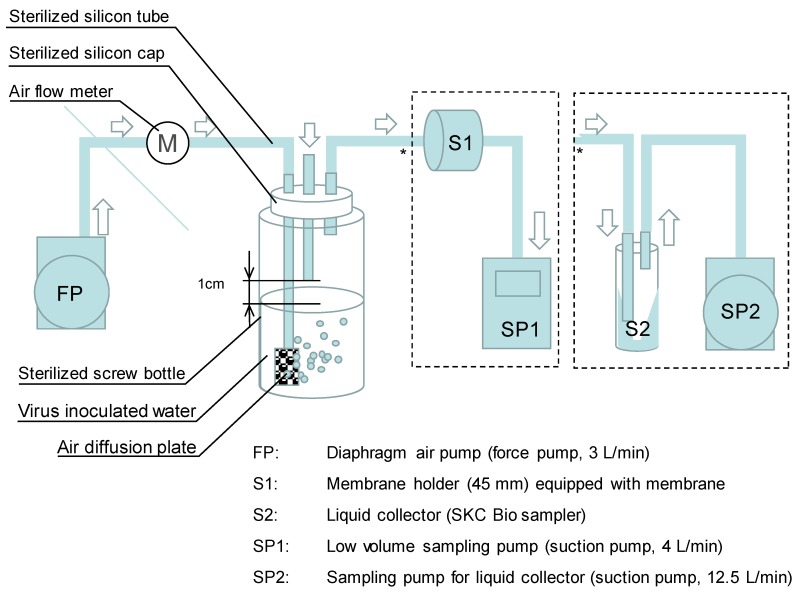
Experimental settings for comparison with the existing sampling method.

A mixed cellulose membrane (HA 0.45 µm, Millipore) was used as collection media with glycine buffer (pH 9.5) to elute the viruses as previously tested among various membrane materials and pore sizes [13]. The membrane was placed in a sterilized 47 mm monitor holder. The HA membrane was proofed to be effective in collecting enteric viruses in the water sample and in eluting the viruses in alkaline solution [14]. The developed method was evaluated by comparing it with an existing liquid collection method (Figure 1). For the liquid collector, we used an SKC Biosampler in which the air was in contact with the liquid circulating inside the container as the standard collection device for viruses among various liquid collectors [12]. The SKC Biosampler was operated with a vacuum pump at a flow rate of 12.5 L/min. In this experiment, two pumps were prepared separately; one for bubbling the viruses and another for sampling such that the airflow rate for bubbling was the same between the newly developed method and the SKC Biosampler. F-specific RNA coliphage Qbeta [15], Poliovirus (LSc-2ab Sabin strain), and murine NV (S7-PP3 strain, isolated in Japan) were used to test the recovery media. The coliphages were propagated in bacterial host *Eschelichia coli* (*E. coli*) K-12 F+ (A/λ) in LB broth solution, followed by filtration with the membrane (pore size 0.45 µm). Poliovirus and murine NV (MNV) were propagated in RAW264.7 cells as previously described [16] to obtain 4.8 × 10^9^–3.4 × 10^11^ plaque-forming units per mL. The titer of the phage and virus stock solution was determined by plaque assay using a double agar overlay method. Then the virus stock solutions (0.1–10 mL) were inoculated into 1 L of sterilized phosphate buffer solution. A 100 mL portion of the inoculated solution was aerated in a 250 mL gas washing bottle by a vacuum pump at a flow rate of 4–12 L/min to generate the virus-containing aerosols. A portable sampling mini-pump (Shibata) and low-volume air sampler (AirCheck HV30) were used for aspiration. The generated aerosols were transported by silicon tubes directly to the collection apparatuses.

### 2.2. qPCR Assay

A 140 µL of the eluate was used for the RNA extraction process by Qa IAamp viral RNA mini kit (Qiagen, Japan) to obtain a final volume of 60 µL. Then the samples were subjected to a reverse transcription step using the High Capacity cDNA RT kit with RNase (Applied Biosystems) following the manufacturer's protocol. Five microliter portions of cDNAs were quantified by real-time quantitative PCR using the ABI PRISM 7500 sequence detection system (Applied Biosystems). The sequence of primers and probes [14,17,18,19] and thermal conditions of real-time PCR are shown in Table S1.

### 2.3. Sampling at the Wastewater Treatment Plant

Air and water samples were collected from a wastewater treatment plant located in Japan with a capacity of 450,000 m^3^/day, adopting the conventional activated sludge (AS) treatment. The treatment process consists of a grit chamber, AS chamber, final settlement chamber, and chlorine contact chamber. The wastewater was mostly made up of domestic sewage since the catchment area of the plant was residential. The plant was located in a residential area and thus all treatment facilities were in the buildings to prevent the odor from getting outside. The AS chamber was covered and equipped with an exhaust duct. The air in the exhaust duct from the AS chamber and the grit chamber was treated with a wet type air scrubber (mist separator) followed by a biological deodorization chamber and an activated carbon deodorization chamber. The treated air was exhausted from an exhaust tower at a public park.

**Figure 2:**
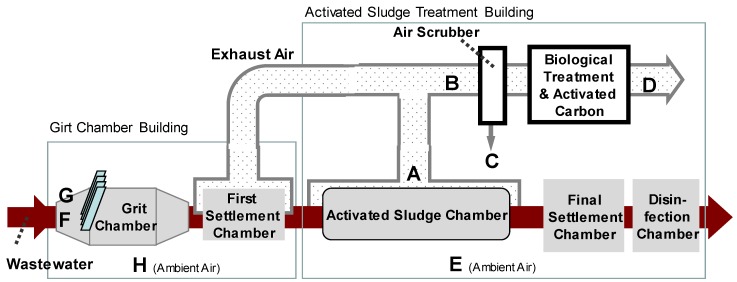
Schematic of wastewater treatment plant treatment flow and sampling points. A; Activated sludge chamber, B; Exhaust air duct, C; Drainage of mist separator, D; Treated air, E; Ambient air at activated sludge building, F; Grit chamber (Floor Level + 0 m near wastewater inflow screen), G; Grit chamber (Floor Level + 1.2 m near wastewater inflow screen), H; Ambient air at grit chamber building.

Figure 2 represents the sampling points at each treatment process. Air at the AS chamber (A) was taken by silicon tube from an inspection hole on top of the chamber cover. The sampling point of the air was approximately 80 cm above the liquid surface of the AS. Air from the exhaust duct (B) and treated air (D) was also taken by silicon tube from an odor inspection hole of the duct. The drain sample from the wet type air scrubber (C) was taken in liquid form. Points F and G were right above the inflow screen of the grit chamber. Point F was at the floor level on the grating while G was sampled at 1.2 m above the ground using a tripod (Figure S1). Ambient air in the AS building (E) and the grit chamber building (H) were also sampled at a 1.2 m height from the ground. Access to points F and G was controlled only for the workers, while points E and H were in the middle of the factory, accessible to visitor tours. There was no other source of droplets or aerosols of wastewater than those at the sampling points. All the sampling points were inside the building where the outside wind did not affect the sampling procedure. The air temperature inside the building was not an extreme condition; the temperature at point A was measured to be 25.0 °C (November 2007), 17.6 °C (December 2007), and 16.5 °C (January 2008). The ambient air temperature was measured at some sampling points for reference purposes, recording 24.2 °C at site E in November 2007 and 13.5 °C at Site F in January 2008.

## 3. Results and Discussion

### 3.1. Evaluation of the Developed Sampling Method

From the three trials for both the developed HA vortex method and the liquid collector, the captured virus amounts showed similar collection capacities; there were no significant differences for captured viruses per m^3^ of air between the methods (Table 1). The recovery ratio was not obtained because the dispersion ratio of viruses from the bubbled water was unknown. MNV tended to be recovered more than EV and bacteriophages (Qβ). The difference in recovery by the virus species may be due to the mechanisms by which viruses were transported from the liquid to air. The tendency for the transportation of the virus from the aqueous phase to air is unclear on the laboratory scale. The previous study showed that the hydrophobicity of the enteric viruses was different among species [20]. The hydrophobic particles are more likely to be aerosolized (transported to air–water interfaces). Results from the field also support that the difference in transportation capacity from seawater to air among various taxa of bacteria was due to different levels of hydrophobicity [21].

### 3.2. Application to Wastewater Treatment Plant

Table 2 shows the results of virus detection from the air and the AS or raw sewage at all sampling points and periods. AdV, NV GI, and NV GII were detected in all water and sludge samples. The detection rate of viruses in the air at sampling points A, B, and F was 89% (8/9) (Table S2). NV GII showed the highest concentration among the viruses tested, with the highest concentration observed at the grit chamber (F, 6.0 × 10^2^ copies/m^3^ in geometric mean, n = 3), followed by the AS chamber (A, 2.4 × 10^2^ copies/m^3^, n = 2).

**Figure 3:**
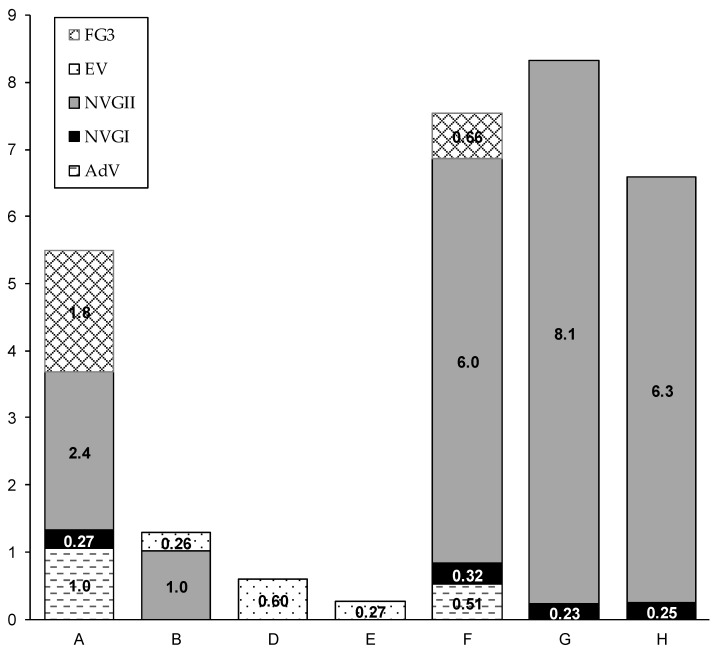
Virus detection at the wastewater treatment plant. Note: All data are shown in geometric means of several sampling trials (see Figure S2 for detailed data). NV GI; Norovirus genogroup I, NV GII; Norovirus genogroup II, AdV; Adenovirus (all serotypes), FG3; F+-specific RNA coliphage serotype 3.

Figure 3 shows the quantified virus concentration at each site. The exhaust air shows high NV GII concentration (1.0 × 10^2^ copies/m^3^, n = 2) before air treatment (B), but NV was not detected after air treatment (D). EV was detected only once from the post-treatment air, though the level was below the quantification limit. Exhaust air treatment effectively reduced the virus in the air, which was also supported by the fact that the viruses were detected from the drain of the air scrubber (Table 2). Viruses (NV GI, NV GII, EV and FG3) were observed in the ambient air in the grit chamber building (H, 6.3 × 10^2^ copies/m^3^). Sampling location H was in the aisle, where there was no machinery or wastewater surface within 2–3 m. The detection of viruses in distant locations such as E and H suggests that the viruses may be aerosolized and dispersed in the building.

Detected locations and sampling periods of NV GI and GII were consistent. For instance, the result of NV GII was always positive if that of NV GI was positive (6/6). Furthermore, NV GI showed the highest concentration at points F and G in December 2017, when the NV GII concentration was the highest. NV GI was quantified in only two samples. On the other hand, AdV was observed in the sample in which NV GII was not detected. In this sample, false-negative results may have been obtained for NV GII because of the problem in the sampling method (see Section 3.4, a comparison with a liquid sampler, for discussion).

High virus concentration at the grit chamber building implies that risk is relatively higher at the place that is in contact with raw sewage, as compared to the location of the treatment process in the AS chamber. Virus aerosols may be supplied from raw water pipes because of the pumping at the upstream of the pipeline, or aerosolized at the mechanical stress at the mechanical screen, which is the location where regular monitoring and maintenance is necessary to precwent garbage from clogging the screen. The risk for WWTP workers is normally controlled because the maintenance personnel usually wear masks and other protective equipment. However, those risk control measures have not been evaluated considering the possible ingestion of enteric viruses from the air. Although our results do not give a comprehensive risk evaluation, they at least show that the protection measures at the grit chamber or near the raw sewage inflow should be prioritized to avoid the unintended ingestion of enteric viruses from sewage.

### 3.3. Comparison of Virus Concentration in the Water and Air 

**Figure 4:**
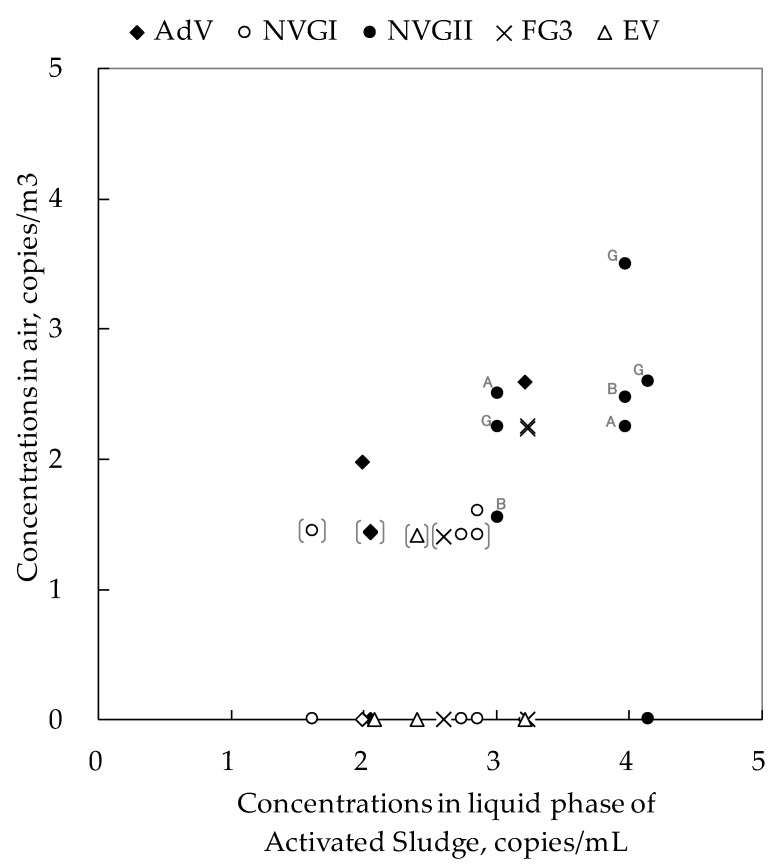
Correlations between virus concentration in activated sludge (liquid phase) and in the air. Notes: The plots on the horizontal axis represent samples not detected. [ ]: Under the quantification limit (detected but not quantified; the plot gives the detection limit value). NV GI; Norovirus genogroup I, NV GII; Norovirus genogroup II, AdV; Adenovirus (all serotypes), FG3; F+-specific RNA coliphage serotype 3.

The virus concentration in the AS was compared with that in the air at sampling points near the wastewater or the AS (A, B, and F) (Figure 4). There was a moderate to strong correlation between the log-transformed virus concentration in the liquid phase of the AS and the virus concentration in the air (r = 0.74, Pearson’s correlation test *p* < 0.001).

The overall correlation between the air and the liquid phase implies that the virus concentration in the air was quantified properly. Given that all conditions in the AS are controlled, the virus concentration in the air should be well correlated with the liquid phase. It is true that the correlation is not always consistent; the NV GII concentration in the AS was higher in January than in December, while the concentration in the air (sampling point A) was lower in January. This result may imply that the virus concentration in the AS was not the only factor to be transmitted through the air. For instance, the strength of aeration in the AS chamber may increase the rate of the virus droplet or aerosol generation. Further study is needed to substantiate the accuracy of the virus detection method in controlled settings in a laboratory experiment.

### 3.4. Comparison with Liquid-Based Sampler

The results in the two sampling periods (December 2007 and January 2008) from the air above the AS chamber (at point A) were compared (Table 3). There are cases in which either our method or the liquid collector was negative while the other was positive.

There was a case in which our method was not as good as the liquid collector. For instance, NV GII was detected by both the HA vortex method and the liquid collector in December 2007, while it was detected only by the liquid collector in January 2008. It was observed at the laboratory that, two to three hours after the sampling, only the membrane sample at site A in January 2008 was wet. The surface of the membrane was obviously wet and its color was changed (slightly transparent). The reason for the wet membrane condition may be due to increased water droplets from the AS caused by the operating conditions, or the aeration intensity of the AS chamber may have been high. The wetness obstructed the membrane’s pores and possibly the air shortcut in the apparatus. Higher humidity in the air may also have affected the collection ratio because humidity moisturizes the membrane surface and may change the electrostatic condition of the membrane. From this result, it should be noted that our collection method may not be stable if the environmental conditions change. Alternatively, the diameter of droplets and aerosols may affect the collection efficiency because the pore size of the membrane was much larger than the viruses. In this sense, a negative result for the virus cannot guarantee the absence of viruses in the air. On the other hand, only our HA vortex method detected NV GI and a higher concentration of NV GII in December 2007. 

The results show that our HA vortex method is comparable to the existing liquid collector concerning the detection of viruses in the air. In addition, the HA vortex method was capable of sampling the air for a longer period than the liquid collector; the liquid collector is normally capable of sampling for only 30 min for fear the liquid will evaporate. Although further study is needed to overcome the false-negative case, it can be easily avoided since it was visibly identifiable by the wet condition of the membrane after sampling. Also, the wet condition of the membrane only happened in a case where samples were taken from an AS chamber with actively aerated water, which thus produced many water droplets. This condition is not likely to arise in ambient air sampling. Therefore, considering sampling ease, our method is superior to the existing method because the sampling media is a solid membrane that can easily be handled and transported. 

## 4. Conclusions

We developed a mobile virus collection method for sampling enteric viruses from the air (HA vortex method), which was optimized for detection by PCR. The method was confirmed to have a similar virus collection ability to that of the existing collection method using liquid media during the laboratory test. Further study is, however, required to improve the collection method since it showed a false-negative result in a field sample when the membrane was wet. The failed case was visibly identifiable due to the membrane surface conditions. To assure the reproducibility of the results, careful checking the membrane condition after the sampling is necessary when the method is applied to a highly humid sampling location. Despite this weakness, the portable detection method we presented has a high potential for the detection of viruses both in the laboratory and the field. The collection media is solid, light, and handy and the method avoids on-site manipulation, which may pose a risk of contamination to the samples. The method has an advantage for WWTP sampling because of the virus-abundant nature of its environment. The developed handy and portable method will encourage the study of enteric viruses from the air, which is an understudied research topic. 

The developed method was applied to a WWTP in Japan and successfully detected enteric viruses in the air which pose an occupational risk for the wastewater treatment plant workers. As the air scrubber removes the viruses dispersed from the WWTP, the risk to neighbors can be controlled by conventional odor control measures. Among all the treatment processes, NV GII was detected in the highest frequency and concentration at the grit chamber. The research suggests that the air near raw sewage has a higher risk of dispersing viruses than the air generated by treatment processes such as AS. It is recommended that appropriate protective measures be taken against the unintended ingestion of enteric viruses from the air, especially near raw sewage.

## Figures and Tables

**Table 1:** Comparison of recovery ratio between developed method (HA vortex) and liquid collector.

Trial	Method	Concentration of Viruses in the Bubbled Water Sample (Copies/mL Water)						Dissipated Water Volume (mL)	Air Volume (m^3^)	Captured Viruses per Air (Copies/m3 Air)		
		Qβ		PV		MNV				Qβ	PV	MNV
		Before	After	Before	After	Before	After					
1	HA vortex	2.9 × 10^8^	2.7 × 10^8^	2.5 × 10^6^	1.6 × 10^6^	7.7 × 10^5^	1.5 × 10^6^	2.06	121	1.3 × 10^2^	2.5 × 10^0^	1.9 × 10^3^
	Liquid Collector							4.33	375	6.7 × 10^1^	2.1 × 10^1^	3.2 × 10^2^
2	HA vortex	3.1 × 10^8^	3.3 × 10^8^	2.4 × 10^6^	1.6 × 10^6^	1.4 × 10^6^	7.2 × 10^5^	2.01	121	5.4 × 10^1^	9.5 × 10^0^	3.9 × 10^2^
	Liquid Collector							3.52	375	4.9 × 10^1^	2.3 × 10^1^	1.6 × 10^2^
3	HA vortex	3.3 × 10^8^	2.9 × 10^8^	2.4 × 10^6^	2.1 × 10^6^	1.4 × 10^6^	7.2 × 10^5^	2.02	121	3.5 × 10^1^	1.2 × 10^1^	3.5 × 10^2^
	Liquid Collector							4.37	375	5.5 × 10^1^	2.1 × 10^1^	2.8 × 10^2^

Note: Qβ; Bacteriophage, PV; Poliovirus, MNV; Murine norovirus, Before/After; Virus concentration of the virus-inoculated water sample before/after bubbling (virus generation) manipulation.

**Table 2:** Virus concentrations in sewage and activated sludge.

Trial	Sample Water Type	Virus Concentration, Copies/mL Water				
		AdV	NV GI	NV GII	FG3	EV
Nov-07	Activated Sludge	1.1 × 10^2^	4.1 × 10^1^	1.0 × 10^3^	1.7 × 10^3^	1.2 × 10^2^
Dec-07	Activated Sludge	9.7 × 10^1^	7.4 × 10^2^	9.4 × 10^3^	4.0 × 10^2^	2.5 × 10^2^
	Drain from Mist Separator	5.2 × 10^-1^	1.7 × 10^0^	1.5 × 10^2^	3.8 × 10^0^	+
Jan-08	Activated Sludge	1.6 × 10^3^	5.5 × 10^2^	1.4 × 10^4^	4.0 × 10^2^	1.6 × 10^3^
Jan-08	Raw Sewage	4.4 × 10^3^	4.1 × 10^2^	2.5 × 10^4^	1.3 × 10^3^	2.5 × 10^3^
Jan-08	Drain from Mist Separator	ND	1.1 × 10^1^	9.4 × 10^2^	1.1 × 10^1^	7.8 × 10^0^

Note: +; Detected but not quantified. NV GI; Norovirus genogroup I, NV GII; Norovirus genogroup II, AdV; Adenovirus (all serotypes), FG3; F+-specific RNA coliphage serotype 3.

**Table 3:** Comparison between the developed method (HA vortex) and liquid collector.

Sampling Period	Site A (Copies/mL)					
	HA Vortex			Liquid Collector		
	NV GI	NV GII	FGIII	NV GI	NV GII	FGIII
December-07	+	1.8 × 10^2^	-	-	+	-
January-08	-		-	-	1.2 × 10^3^	3.1 × 10^3^

Note: + Detected but not quntified.

## Supplementary Materials

The following are available online at https://www.mdpi.com/2076-0817/8/3/131/s1, Table S1: Sequences of primers and probes for real-time PCR, Table S2: Detection of viruses from the wastewater treatment plant (detail), Figure S1: Photo of sampling (at the wastewater treatment plant).
