# Supplementary material for: Development of a Portable Detection Method for Enteric Viruses from Ambient Air and Its Application to a Wastewater Treatment Plant
Source: Pathogens. 2019 Aug 24;8(3):131. doi: 10.3390/pathogens8030131 (PMC6789845; doi:10.3390/pathogens8030131)
Supplement: Supplementary file 1 [file pathogens-08-00131-s001.pdf]

TableS1 Sequences of primers and probes for real-time PCR

Norovirus Genotypes I (Kageyama et al. 2003)

Name (Function*)	Sequence (5' → 3')	Tm	Final
COG1F(+)	CGYTGGATGCGNTTYCATGA	61.1	400nM
COG1R(−)	CTTAGACGCCATCATCATTYAC	56.3	400nM
RING1(a)-TP(P)	FAM-AGATYGCGATCYCCTGTCCA-TAMRA	61.7	300nM

Thermal conditions: 95℃ 15sec → 56℃ 60sec [85bp]

Norovirus Genotypes II (Kageyama et al. 2003)

Name (Function*)	Sequence (5' → 3')	Tm	Final
COG2F(+)	CARGARBCNATGTTYAGRTGGATGAG	65.6	400nM
COG2R(−)	TCGACGCCATCTTCATTCACA	61.1	400nM
RING2-TP(P)	FAM-TGGGAGGGCGATCGCAATCT-TAMRA	65.8	300nM

Thermal conditions: 95℃ 15sec → 56℃ 60sec [98bp]

Enterovirus (Katayama et al. 2002)

Name (Function*)	Sequence (5' → 3')	Tm	Final
Pan-Entero(+)	CCTCCGGCCCCTGAATG	60.9	400nM
Pan-Entero(−)	ACCGGATGGCCAATCCAA	60.6	400nM
Pan-Entero(P)	FAM-CCGACTACTTTGGGTGTCCGTGTTTC-TAMRA	66.3	300nM

Thermal conditions: 95℃ 15sec → 60℃ 60sec [197bp]

Adenovirus for all serotypes (Heim et al. 2003)

Name (Function*)	Sequence (5' → 3')	Tm	Final
AQ1(+)	GCCCCAGTGGTCTTACATGCACATC	66.4	500nM
AQ2(−)	GCCACGGTGGGGTTTCTAAACTT	63.2	500nM
AP(P)	FAM-TGCACCAGACCCGGGCTCAGGTACTCCGA-TAMRA	78.1	400nM

Thermal conditions: 95℃ 3sec → 55℃ 10sec → 65℃ 60sec [132bp]

Murine norovirus 1 (Kitajima M. unpublished)

Name (Function*)	Sequence (5’→3’)	Tm	Final
MKMNVF(+)	CGGTGAAGTGCTTCTGAGGTT	58	400nM
MKMNVR(−)	GCAGCGTCAGTGCTGTCAA	58	400nM
MKMNVP(P)	FAM-CGAACCTACATGCGTCAG-MGB	68	300nM

Thermal conditions: 95℃ 15s→56℃ 60s [60bp]

F-specific RNA coliphage serogroup 3 (Ogorzaly et al. 2006)

Name (Function*)	Sequence (5’→3’)	Tm	Final
FG3(+)	CCGCGTGGGGTAAATCC	-	400nM
FG3(−)	TTCTTACGATTGCGAGAAGGCT	-	400nM
FG3(P)	FAM-AAGCGGGTGCAGTT-MGB	-	300nM

Thermal conditions: 95℃ 15s→60℃ 60s [116bp]

* +; Forward primer, −; Reverse primer, P; Probe

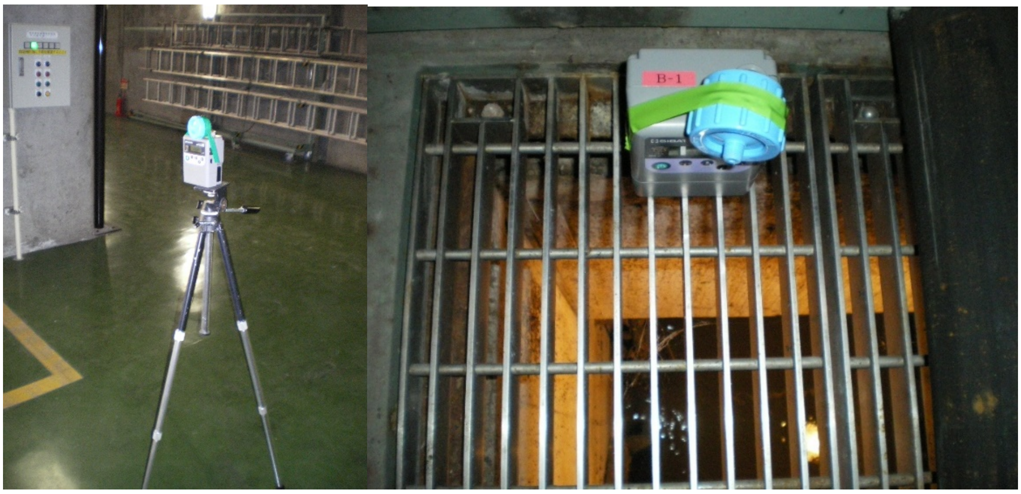

Figure S1 Photo of Sampling (at Wastewater Treatment Plant)

Table S2 Detection of Viruses from the Wastewater Treatment Plant (Detail)

Sampling Time	Sampling Location	AdV copies/m^3 air	NV GI copies/m^3 air	NV GII copies/m^3 air	FG3 copies/m^3 air	EV copies/m^3 air
Nov-07	A	+	+	3.2E+02	1.8E+02	−
	B	−	−	3.5E+01	−	−
	D	−	−	−	−	+
	E	−	−	−	−	+
	F	+	−	1.8E+02	1.7E+02	−
	G	NA	NA	NA	NA	NA
	H	NA	NA	NA	NA	NA
Dec-07	A	−	+	1.8E+02	−	−
	B	−	−	3.0E+02	−	+
	D	−	−	−	−	−
	E	−	−	−	−	−
	F	9.6E+01	4.0E+01	3.1E+03	+	−
	G	−	2.3E+01	8.1E+02	−	−
	H	NA	NA	NA	NA	NA
Jan-08	A	4.0E+02	−	−	−	−
	B	−	−	−	−	−
	D	NA	NA	NA	NA	NA
	E	NA	NA	NA	NA	NA
	F	−	+	4.0E+02	−	−
	G	−	−	−	−	−
	H	−	+	6.3E+02	−	−

−; Not Detected, +; Detected, NA; Not Analyzed

NV GI; Norovirus Genogroup I, NV GII; Norovirus Genogroup II, AdV; Adenovirus (all serotypes), FG3; F+ specific RNA Coliphage serotype 3
